# A study of chloride binding capacity of concrete containing supplementary cementitious materials

**DOI:** 10.1038/s41598-024-62778-6

**Published:** 2024-06-05

**Authors:** Heba Abd El-Fattah, Yehia Abd El-Zaher, Mohamed Kohail

**Affiliations:** Structural Engineering Department, Faculty of Engineering, Ain Shams University, Cairo, Egypt

**Keywords:** Chloride binding, Seawater, Sea-sand, Steel corrosion, Concrete durability, Supplementary cementitious materials, Highly aluminates materials, Friedel’s salt, Civil engineering, Structural materials

## Abstract

Chloride-induced steel corrosion is known to be a very common kind of deterioration of reinforced concrete. It is beneficial to bind free chloride ions to reduce the corrosion probability of the reinforcement embedded in the concrete. The binding capacity of the concrete varies according to its cementitious system. This paper investigates the chloride binding capacity of different kinds of supplementary cementitious materials (SCMs): Ground granulated blast furnace slag (GGBFS), Fly ash, and Metakaolin as a partial replacement of Ordinary Portland Cement (OPC). Different properties of concrete after chloride binding are assessed by carrying out the following tests: half-cell potential, accelerated corrosion test, compressive strength, rapid chloride penetration test, sorptivity test, measuring pH value of concrete, and XRD. The results showed that utilizing the SCMs in concrete can enhance the chloride binding capacity, especially those materials that have high quantities of aluminate and calcium in their chemical composition like GGBFS. Based on testing results, it’s recommended that the limit of the chloride content in the different codes should be revised regarding the binding capacity according to the type and quantity of the cementitious materials used.

## Introduction

The durability of reinforced concrete is known to be greatly affected by steel corrosion which may happen due to carbonation and/or the presence of chloride ions in concrete^1–5^. Chlorides can get into the concrete from the surrounding environment, especially in marine and coastal regions or they may be included in any of the concrete ingredients during the concrete manufacturing process. Because of the high effect of chloride ions on the corrosion of steel rebars, standard codes have set restrictions on the chloride content in reinforced concrete^6^. Free chloride ions in concrete cause pitting corrosion of steel rebars but a part of chlorides existing in concrete is bound physically and/or chemically to the hydration products that result from the reaction of cement with water. And these bound chlorides almost have no risk of steel corrosion^7–11^. The chloride binding capacity of the concrete varies according to the type and content of the binder so, choosing a proper cementitious material with a high chloride binding capacity in reinforced concrete is very beneficial. The mechanism of the chemical binding of free chloride ions in concrete is simply attributed to the formation of calcium chloro aluminate which is commonly known as Friedel’s salt that results from the chemical reaction between the free chloride ions and tricalcium aluminate^7–9,12–15^ as shown in Eq. (1):

1 $$\begin{gathered} {\text{C}}_{3} {\text{A}} + {\text{Ca}}\left( {{\text{OH}}} \right)_{2} + 2{\text{Cl}}^{ - } + 10{\text{H}}_{2} {\text{O }} \to {\text{ C}}_{3} {\text{A}}.{\text{CaCl}}_{2} .10{\text{H}}_{2} {\text{O}} + { }2{\text{OH}}^{ - } \hfill \\ \quad \quad \quad \quad \quad \quad \quad \quad \quad \quad \quad \quad \quad \quad \quad \quad ({\text{Friedel}}^{\prime}{\text{s}}\,{\text{salt}}) \hfill \\ \end{gathered}$$

So, the chemical binding of chloride ions (formation of Friedel’s salt) which is more stable than the physical binding is proved to be mainly related to the availability of both calcium and aluminate phases in the concrete^10^. That is why sulfate-resisting Portland cement (SRPC) can’t bind chlorides effectively^16^.

Fly ash (FA), metakaolin (MK), and ground granulated blast furnace slag (GGBFS) are proved to be the most common SCMs that can bind chlorides effectively as they have Al_2_O_3_ much more than OPC as the following^15,17^:

**Table:** 

Binder	Al_2_O_3_ range (%)
OPC	3–8
GGBFS	14–18
FA	10–30
MK	35–50

Therefore, using cementitious materials as concrete binders which include high aluminate content is considered the key to enhancing the chloride binding capacity of concrete^2,3,5,8,9,13,15,17–21^. But Mulbah et al*.*^13^*,* as well as Thomas et al*.*^15^*,* have found that in some cases, increasing the amount of C_3_A in the cementitious materials does not necessarily lead to increasing the chloride binding capacity. Also, the w/c ratio was found to have a positive effect on enhancing the chloride binding capacity in concrete^15,16^. Other factors affecting the chloride binding capacity in concrete, such as hydration age, curing temperature, and cation type engaged with chloride have been studied^16,22^*.*

The utilization of supplementary cementitious materials (SCMs) in the concrete industry has increased incredibly during the last few decades as they greatly enhance the physical and mechanical properties of the concrete^23–29^. Besides, some of the SCMs have a great role in chloride binding which contributes to protecting the reinforcing steel from corrosion^1,3,17,22,30,31^. Dhir et al*.*^8^ studied the effect of different replacement levels of GGBS in pastes and sodium chloride solution with different concentrations were added to the powdered specimens after hydration. The research stated that the chloride binding capacity increased as the replacement level increased for all concentrations of chlorides due to the increase in the alumina content in the pastes.

Thomas et al.^15^ investigated the impact of alumina-rich pozzolanic materials on the chloride binding in hardened cement pastes. The research highlighted that the higher the percentage of alumina the higher the chloride binding capacity.

It was reviewed by Shi et al.^4^ that SF reduces the chloride binding capacity of concrete for a group of reasons. Firstly, SF reduces the alkalinity of the pore solution because of the reaction between silicon dioxide and the portlandite which leads to an increase in Cl^−^/ OH^−^ and this is also asserted by other studies^32,33^, secondly, the chemical composition of SF almost has no alumina then, there is no chance for chemical binding to occur.

It was also reviewed by the same research^4^ that the effect of GGBFS partial replacement on the chloride threshold is still debatable additionally, adding GGBFS in concrete not only refined its microstructure and enhanced later-age strength but also reduced the chloride diffusivity and increased both corrosion resistance and the chloride binding capacity by the formation of Friedel’s salt and the physical adsorption.

Page et al.^34^ replaced 30% of OPC with fly ash and found that it was effective in chloride binding and corrosion resistance and this can be explained as the alumina phase in fly ash contributed to the formation of insoluble-complex salt (Calcium-chloro-aluminate hydrate) which is called as Friedel’s salt.

There is quite limited knowledge regarding the impact of the chloride binding process using different SCMs on the mechanical and physical properties of reinforced concrete and its corrosion behavior after binding. In this paper, the chloride binding capacity of different SCMs (used as a partial replacement of OPC) was investigated in addition to clarifying the effect of such a process on the corrosion behavior of steel embedded in concrete.

The experimental program of this research included the determination of the chloride binding capacity of cement pastes mixed with two ratios of chloride content (0.4 and 0.8% from the binder weight) and three types of SCMs (Fly ash, GGBFS, and Metakaolin). To measure the chloride binding in concrete, many methods have been conducted like titration with silver nitrates which is considered the most common method because of its ease from the practical point of view, besides, it gives results with accepted accuracy^7,11,13,16,20^. Also, XRD and TGA are widely used to examine the chemical binding by showing the peak of Friedel’s salt which represents to a great extent the chloride binding in concrete^5,10,12,13,15,35^. Empirical relations (equilibrium method) are used to express the chloride binding capacity when the concrete samples are exposed to an external solution of NaCl of a known concentration^8,13–15,17^. The experimental program of this research includes the assessment of the different properties of concrete mixes that are correspondent/identical to the mixes of previous pastes.

For this approach, the chloride binding capacity of each binder type is examined by measuring the residual-free chloride content in the pastes using the titration method. Half-cell potential test was carried out to investigate the probability of steel corrosion that may happen due to the content of free chloride ions remaining in the concrete after binding. The accelerated corrosion test was conducted to compare the corrosion behavior of steel rebars in each mix. Also, the mechanical properties and durability of concrete were assessed by carrying out the compressive strength test, pull-out test, rapid chloride penetration test, sorptivity test, measuring the pH value of concrete, and XRD.

## Materials and experimental procedure

### Materials

Ground granulated blast furnace slag originated in India, and commercially available fly ash (type F) are used as a partial replacement for OPC^36,37^ in addition, metakaolin that produced by calcining kaolin at 800 °C^38^. The chemical composition of used materials is shown in Table 1. The physical properties of the used well-graded aggregate are shown in Table 2. 100% pure NaCl was added to potable water (containing no considered chloride content) as a percentage of the binder.

**Table 1:** Chemical composition of cementitious materials.

Oxides (%)	CaO	SiO_2_	Al_2_O_3_	SO_3_	Fe_2_O_3_	MgO	Na_2_O	K_2_O	Cl	MnO	TiO_2_	P_2_O_5_	ZrO_2_	Nb_2_O_5_	SrO	LOI^a^
Cement	61.45	19.31	4.59	3.35	3.12	2.59	0.28	0.2	0.05	–	–	–	–	–	–	3.73
GGBFS	36.87	35.4	17.4	0.24	1.4	6.83	–	–	–	0.35	0.11	–	–	–	–	0.5
FA	1.05	61.65	26.96	–	7.37	1.46	0.38	1.65	0.02	–	–	–	–	–	–	0.36
MK	0.16	57.1	38.1	0.08	0.61	0.23	–	0.03	–	–	1.35	0.08	0.05	0.01	0.01	2.11

^a^LOI, Loss on ignition.

**Table 2:** Physical properties of aggregate.

Property	Specific gravity	Volumetric weight (gm/cm^3^)	% of fine materials	% of water absorption	Chloride ion content (% by weight)	Sulfate content (% by weight)
Fine aggregate (Sand)	2.62	1.61	2.6	–	0.03	0.21
Coarse aggregate (Crushed stones)	2.677	1.531	0.7	2.2	0.015	0.18

### Mixtures proportions

#### Cement pastes proportions

Eighteen mixes of cement pastes were poured with a constant water to binder ratio in all mixes that equal 0.5, and chlorides ratios (as a percentage from the binders’ weight) are chosen to be equal to 0.4% and 0.8%. Table 3 shows the details of the mix proportion for each paste of quantity equal to 250 cm^3^.

**Table 3:** Mixes proportions of cement pastes.

Mix no	OPC (g)	Fly ash (g)	GGBFS (g)	Metakaolin (g)	Water (g)	NaCl (g)	W/B	Cl^− (%)^	Replacement level
1	350	0	0	0	175	2.33	0.5	0.4	–
2	350	0	0	0		4.67		0.8	–
3	297.5	52.5	0	0		2.33		0.4	15%
4	297.5	52.5	0	0		4.67		0.8	15%
5	227.5	122.5	0	0		2.33		0.4	35%
6	227.5	122.5	0	0		4.67		0.8	35%
7	297.5	0	52.5	0		2.33		0.4	15%
8	297.5	0	52.5	0		4.67		0.8	15%
9	227.5	0	122.5	0		2.33		0.4	35%
10	227.5	0	122.5	0		4.67		0.8	35%
11	332.5	0	0	17.5		2.33		0.4	5%
12	332.5	0	0	17.5		4.67		0.8	5%
13	315.0	0	0	35.0		2.33		0.4	10%
14	315.0	0	0	35.0		4.67		0.8	10%
15	297.5	0	0	52.5		2.33		0.4	15%
16	297.5	0	0	52.5		4.67		0.8	15%
17	227.5	0	0	122.5		2.33		0.4	35%
18	227.5	0	0	122.5		4.67		0.8	35%

#### Concrete mixes proportions

All the concrete mixes are identical to that of the pastes but with the addition of fine and coarse aggregate. The mix proportions for 1m^3^ of concrete are shown in Table 4.

**Table 4:** Mixes proportions of concrete (for 1m^3^).

Mix no	OPC (Kg)	Fly ash (Kg)	GGBFS (Kg)	Metakaolin (Kg)	Coarse agg. (Kg)	Fine agg. (Kg)	Water (Kg)	NaCl (Kg)	W/B	Cl^− (%)^	Replacement level
1	350	0	0	0	1117	558	175	1.4	0.5	0.4	–
2	350	0	0	0	1234	616		2.8		0.8	–
3	297.5	52.5	0	0	1474	317		1.4		0.4	15%
4	297.5	52.5	0	0	1474	317		2.8		0.8	15%
5	227.5	122.5	0	0	1465	320		1.4		0.4	35%
6	227.5	122.5	0	0	1465	320		2.8		0.8	35%
7	297.5	0	52.5	0	1479	297		1.4		0.4	15%
8	297.5	0	52.5	0	1479	297		2.8		0.8	15%
9	227.5	0	122.5	0	1476	273		1.4		0.4	35%
10	227.5	0	122.5	0	1476	273		2.8		0.8	35%
11	332.5	0	0	17.5	1478	310		1.4		0.4	5%
12	332.5	0	0	17.5	1478	310		2.8		0.8	5%
13	315.0	0	0	35.0	1475	306		1.4		0.4	10%
14	315.0	0	0	35.0	1475	306		2.8		0.8	10%
15	297.5	0	0	52.5	1471	302		1.4		0.4	15%
16	297.5	0	0	52.5	1471	302		2.8		0.8	15%
17	227.5	0	0	122.5	1458	286		1.4		0.4	35%
18	227.5	0	0	122.5	1458	286		2.8		0.8	35%

### Preparation of specimens

Pastes were cast in cubic molds of size 50 × 50 × 50 mm and de-molded after 24 h then, cured for 28 days at 20 °C by being covered with wet burlap for 28 days at 25 °C and the burlaps were to be wet periodically. For concrete specimens, Table 5 presents the number of specimens needed for testing. All concrete specimens were cured for 28 days at 25 °C in water tanks except specimens used in half-cell potential and accelerated corrosion tests which were cured by being covered with wet burlap.

**Table 5:** Concrete testing specimens.

Test	Specimen dimension (mm)	No. of specimens
Compressive strength	Cube 100 × 100 × 100	6
Pull-out	Cylinder 100 × 200	3
Half-cell potential + Accelerated corrosion	Cylinder 100 × 200	3
Sorpitivity + Rapid chloride penetration + pH + XRD	Cylinder 200 × 100	2

### Testing procedures

#### Determination of free chloride content

The paste cube was crushed, and 20 gm was taken as the test sample. First, the sample was dried for 2 h at 65 °C in the drier then, it was ground to be a very fine powder. 10 gm was taken from the sample and put into a conical and 100 ml of boiled distilled water was added to it and they were put on a magnetic stirrer for 30 min. Secondly, the sample was filtered using filtration paper, and 20 gm of the filtrate was taken for the titration process. Two drops of phenolphthalein were added as a pH indicator, so the filtrate color turned to pink (alkaline medium). Then the solution was neutralized by adding drops of dilute sulfuric acid till the solution would be colorless. Ten drops of potassium chromate were added as an indicator then, adding drop by drop of silver nitrate with a concentration of 0.02 mol/L using a clean pipette. Finally, calculate the amount of silver nitrate consumed by the solution to form a red precipitate. The free chloride content could be calculated as shown in Eq. (2)^7,20^:

2 $$Cl^{ - } = \frac{{\left( { N \times V } \right)_{{{\text{AgNo}}_{{3}} }} \times 0.0355 \times V_{w } }}{{V_{f} \times W_{s} }} \times 100$$

where,

Cl^−^: The amount of free chloride content in the sample as a percentage of the binders (%).

N: The concentration of silver nitrate (mol/l).

V: The volume of silver nitrate consumed in the titration process (ml).

V_w_: The volume of water added to the powdered sample (ml).

V_f_: The volume of filtrate used (ml).

W_s_: The weight of the powdered sample (gm).

The chloride binding capacity (CBC) can be calculated by knowing the free chloride content remained in the paste as shown in Eq. (3):

3 $$CBC= \frac{Total \, chloride-free \, chloride}{Total \, chloride} \times 100$$

where the total chloride content is the known amount of chloride added to the cement paste.

#### Half-cell potential

**Figure 1:**
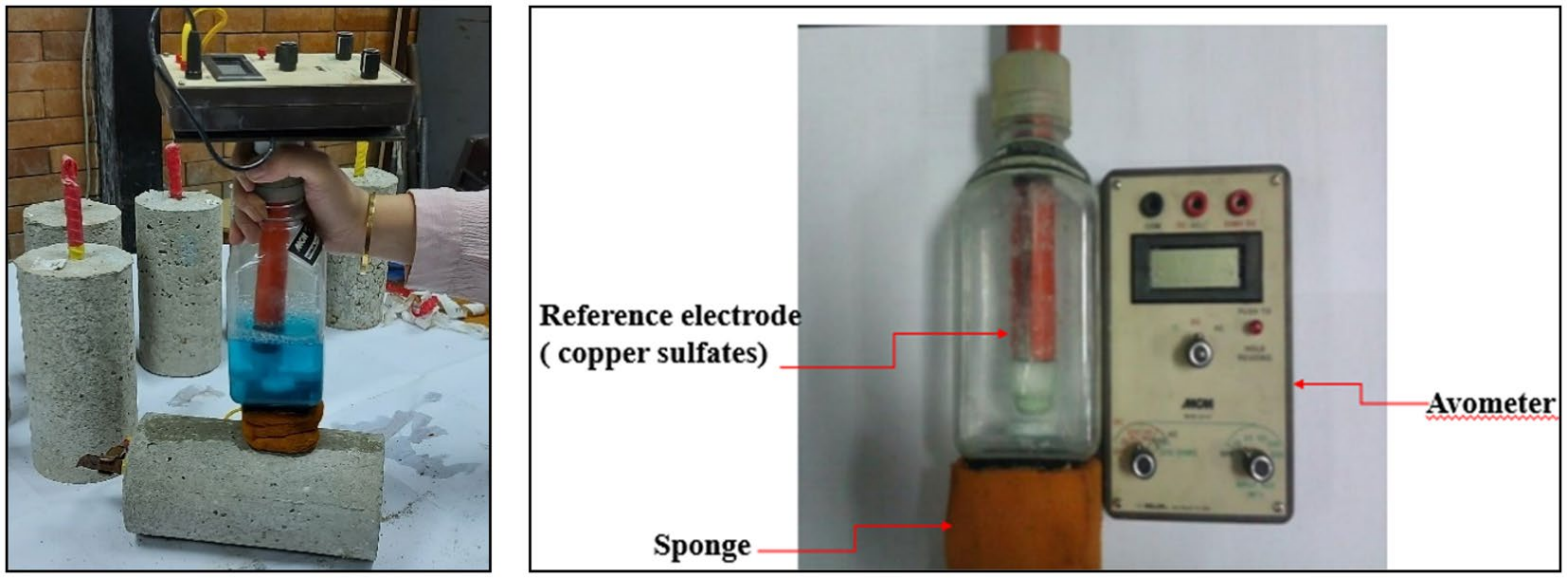
The performed half-cell potential test.

Before carrying out the accelerated corrosion test, a half-cell potential test was done according to ASTM C876^39^ on the lollipop specimens to investigate the probability of corrosion of the steel rebar that may happen due to the amount of free chloride ions that remained in the concrete after binding. The steel rebar was connected to the positive terminal of the voltmeter while the reference electrode (Copper/Copper sulfate) was connected to the negative terminal and the potentials were recorded. The actual test performed is indicated in Fig. 1.

#### Accelerated corrosion

**Figure 2:**
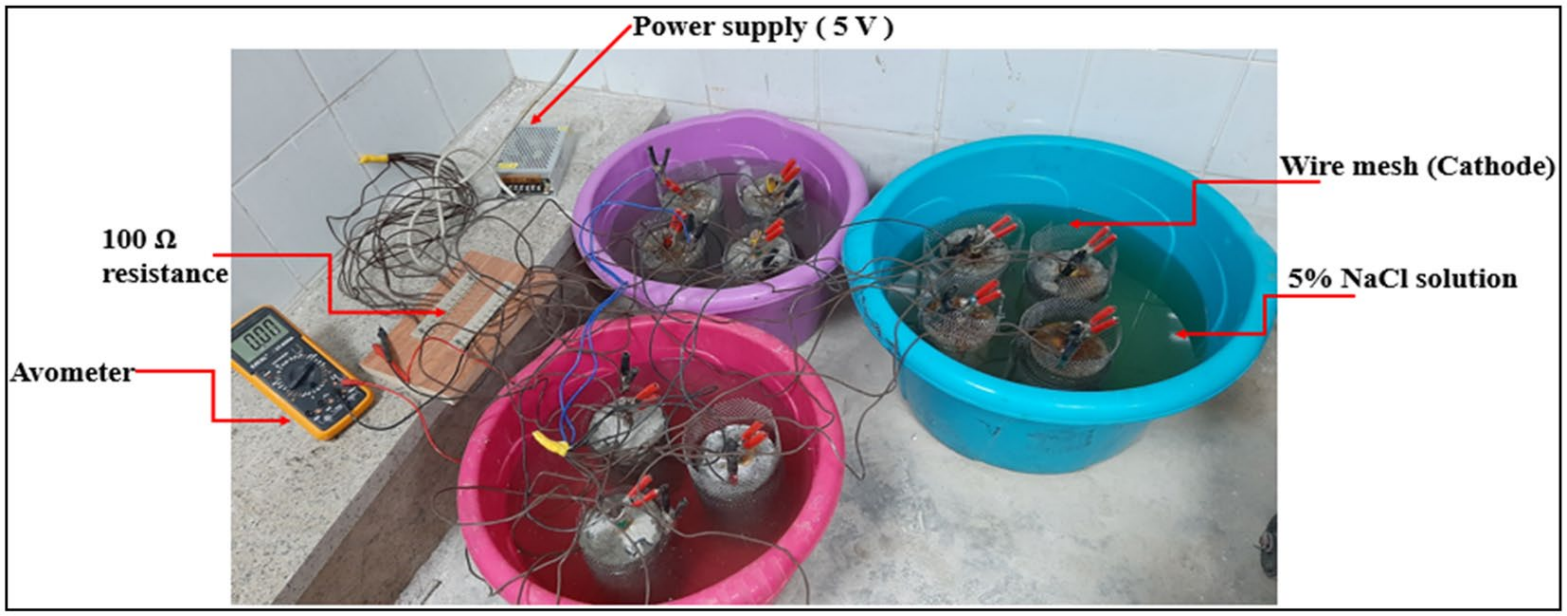
Accelerated corrosion test setup.

An accelerated corrosion test (Impressed voltage technique) was carried out to compare the behavior of the concrete mixes with different binders while exposing them to a corrosion current^20,21,30,40^. Test specimens were concrete cylinders with a centrally embedded steel rebar (lollipop specimen) ensuring a cover of 50 mm and each specimen was surrounded with a wire mesh to work as the cathode. The NaCl solution is of 5% concentration and the DC power supply gave a constant 5 V to initiate the process of corrosion. The value of the electrical current of each specimen was recorded daily and the test setup can be shown in Fig. 2.

#### Mechanical properties

For all concrete mixes, the compressive strength was determined at the age of 7 and 28 days by conducting the compression test to assess the effect of both the existence of NaCl salt in the concrete and the chloride binding process on the compressive strength of the concrete made with different types of binders. The test was carried out on cubic specimens with a length of 100 mm^3,7,12,18,20^.

**Figure 3:**
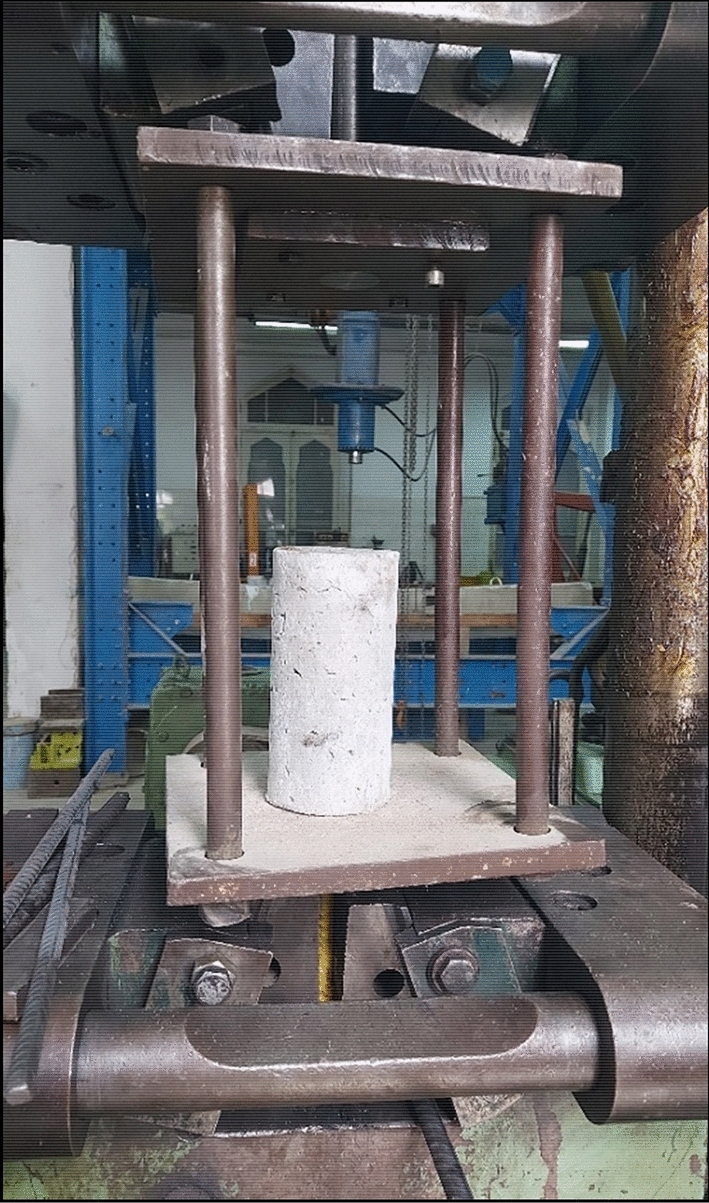
Test setup of pull-out test.

The bond strength between steel and concrete admixed with salt and different binders was evaluated at the age of 28 days through the pull-out test ^18,20^. The test was implemented on cylindrical concrete specimens of 200 mm in height and 100 mm in diameter with entire ribbed steel rebar of 12 mm in diameter embedded in the center of the cylinder. The specimen was fixed in the tensile testing machine with a capacity of 100 tons where the free end of the embedded rebar was pulled out from the concrete as shown in Fig. 3. The bond strength can be calculated from Eq. (4) as shown:

4 $${F}_{Bond}= \frac{P}{\pi d L}$$

where,

P: Failure load.

d: Bar diameter.

L: Bond length between steel and concrete.

#### Rapid chloride ion penetration test

A rapid chloride penetration test (RCPT) was carried out to measure the electrical conductivity of the concrete by measuring the amount of chlorides that can travel through the concrete. The test was performed according to ASTM C1218^41^.

The current (in Amperes) transported across the specimen is plotted versus the time (in Minutes) and the total charge passed (in coulombs) is to be calculated by the trapezoidal rule as shown in Eq. (5):

5 $$Q=900 \left( {I}_{0}+2{I}_{30}+2{I}_{60}+\dots 2{I}_{300}+2{I}_{330}+{I}_{360} \right) (Coulombs)$$

Then, the value of the total charge indicates the chloride ion penetrability and the quality of the concrete.

#### Sorptivity

This test was implemented according to ASTM C1585-04 ^42^ to get the rate of water absorption by capillarity through concrete specimens. The test specimen is a concrete slice of dimensions 100 ± 6 mm in diameter and 50 ± 3 mm in height. The test was performed after 28 days on specimens cured in water tanks. Before testing, the specimens were dried in a drying oven at 100 °C until their weights became constant. The side surface of the specimens was sealed by electrical tape and the top surface was covered by an aluminum sheet to prevent evaporation of the water absorbed from the upper surface. The initial weight of the specimen was recorded then, the specimen was placed on supports where 1–3 mm from its lower surface could be submerged in water.

The weight of the specimen was measured at specific time intervals as shown in Table 6. The specimen should be wiped with a piece of cloth to remove any excess drops of water before being weighed and it should be put above the balance on its top-dry surface.

**Table 6:** Times intervals for recording specimen weight.

Time	
Day	Second
	0
	60
	300
	600
	1200
	1800
	3600
	7200
	10,800
	14,400
	18,000
	21,600
1	92,220
2	193,200
3	268,500
4	432,000
5	527,580
6	622,200
7	691,200

Then, the absorption is calculated from Eq. (6):

6 $$I= \frac{{M}_{t}}{a/d}$$

where,

I: The absorption (mm),

M_t_: the change in specimen mass in grams, at the time t,

a: the exposed area of the specimen, in mm^2^, and.

d: the density of the water in g/mm^3^.

Then, the relation between the absorption and the square root of time is plotted to get both initial and secondary rates of absorption.

#### Determination of pH

The pH value of concrete mixes was measured according to Abdel-Gawwad et al. recommendations ^43^ by grinding concrete to be in a powdered form then, 5 gm of powdered sample was taken with 100 ml of distilled water and put on a magnetic stirrer for 30 min then the solution was filtered and the pH was measured using a digital pH meter.

#### XRD

The XRD test was performed on specified concrete mixes (1,3,7,9,11,15) to identify the main chemical phases existing in them, especially Friedel’s salt (C_3_A.CaCl_2_.10H_2_O) which manifests at position equals 11 (2 ${\varvec{\theta}}$) approximately. The factors on which mixes have been chosen for the XRD test are: 1-Having constant chloride content which was 0.4% as this is the chloride limit stated in the Egyptian code of practice. 2-Having constant and medium replacement ratio of SCMs which is 15% a (Mixes: 3,7, 15). 3-Having high chloride binding capacity to prove the formation of Friedel’s salt in such mixes (Mixes: 9, 11).

The identification of the most probable phases is carried out using a PANalytical computer-certified program with the aid of the International Center of Diffraction Database (ICDD) received with the X-ray diffraction equipment.

## Results and discussion

### Determination of chloride binding capacity

**Figure 4:**
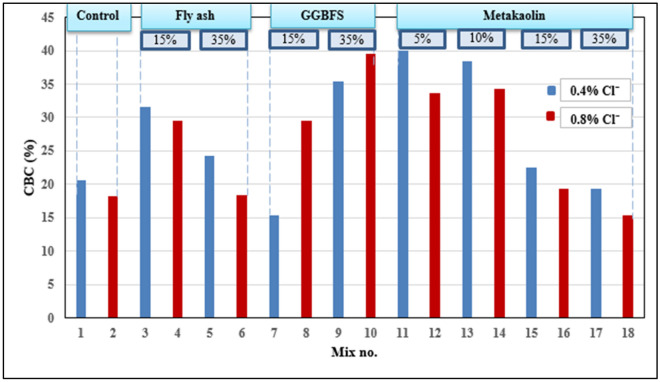
The chloride binding capacity of cement pastes.

The percentages of chloride binding capacity of each mix are calculated based on Eqs. (1) and (2) and the results are presented in Fig. 4. The results indicate a general trend that the chloride binding capacity of mixes containing 0.4% Cl^−^ is higher than that of mixes containing 0.8% Cl^−^ (except for mixes containing GGBFS) which means that the chloride binding capacity decreased by increasing the chloride content in cement pastes. This phenomenon can be explained as the process of chloride binding has a maximum limit depending on the alumina available for the reaction so,—referring to Eq. (2)—by increasing the dosage of chlorides, the total chloride content increases while the bound chlorides remain constant consequently, the resultant chloride binding capacity decreases.

Generally, the results show that the mineral admixtures are effective in being used as a partial replacement of OPC to bind free chlorides as most mixes have chloride binding capacity higher than control mixes which contain only OPC, and this is mainly attributed to the fact that FA, GGBFS, and MK contain more alumina than OPC, so when they are blended with OPC, they can bind more chlorides than OPC can do alone^1,3,4,15^.

For GGBFS mixes, the chloride binding capacity increases with increasing the replacement level because GGBFS has both alumina and calcium needed for chemical binding, that’s why (35% GGBFS) has high chloride binding capacity. While the opposite occurs with mixes containing FA and MK as they almost have no calcium so it becomes harder to form Friedel’s salt^13,17,19,31^ so, (5% MK) has the highest chloride binding capacity among other mixes containing MK.

The mixes that showed the highest binding capacity are 35%GGBFS and 5% MK (mixes 9,10,11, and 12). By studying the binding of these mixes beside the binding capacity of OPC with 0.4% Cl^−^ (mix 1), it can be proposed that the allowable limit of chloride ion content can be increased to be about 0.5% from the binder weight instead of 0.4% for such mixes containing pozzolanic materials as a partial replacement of OPC. And this higher limit will not cause corrosion of steel as confirmed by the results of the half-cell potential test that will be presented later.

### Compressive strength

**Figure 5:**
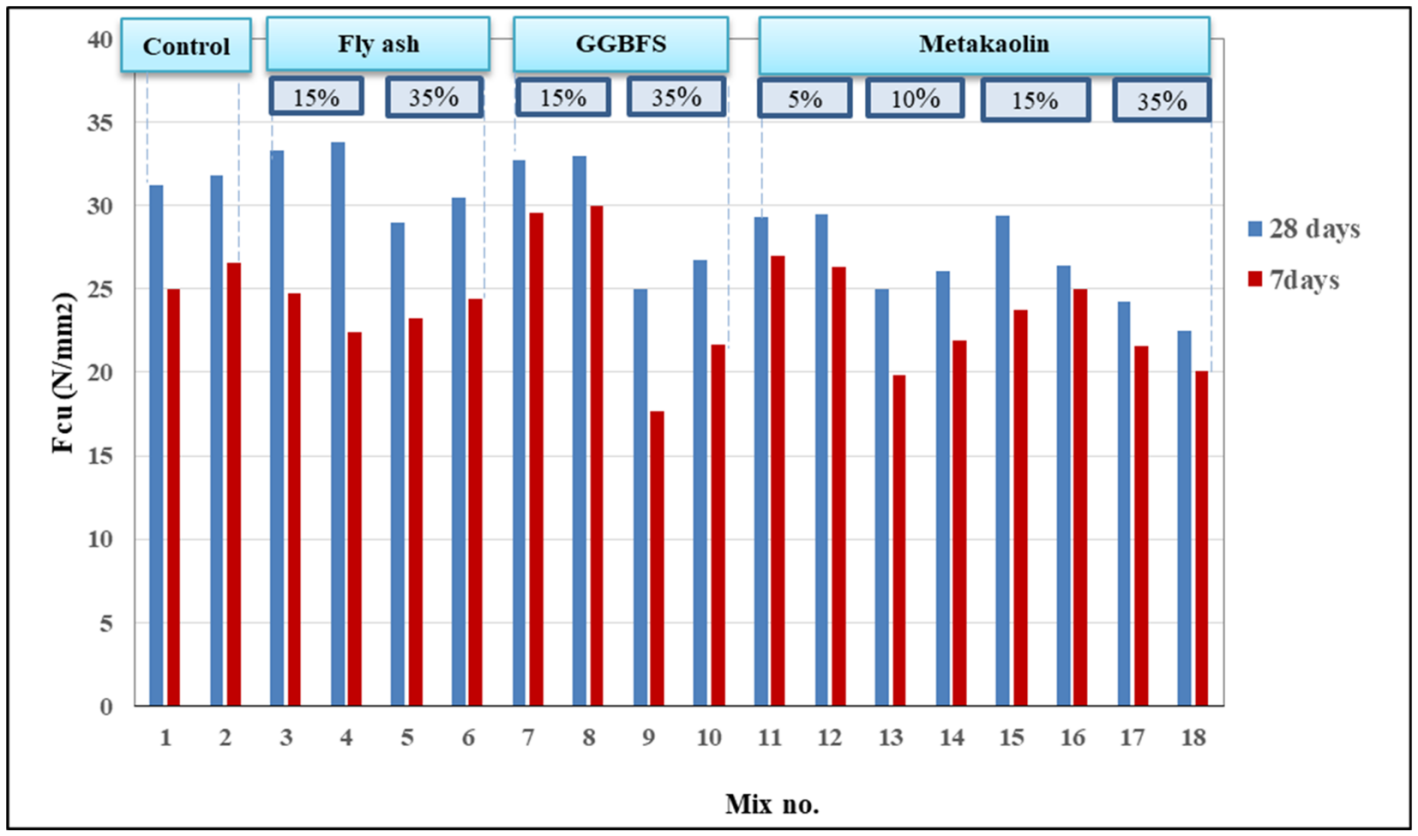
Compressive strength of concrete mixes.

For all concrete mixes, the compressive strength was determined at age 7 and 28 days to evaluate the mechanical properties as shown in Fig. 5. The range of the compressive strength at age 28 days for all mixes is approximately between 23 and 35 N/mm^2^ taking into consideration that pozzolanic materials gain their strength at later ages.

The results of most of the mixes also indicate that mixes with 0.8% Cl^−^ have compressive strength slightly higher than that with 0.4% Cl^−^ which agrees with the literature, and this can be referred to the fact that more chlorides can fill more voids in the concrete, therefore, densify its microstructure which enhances the mechanical properties. So, it could be concluded that the existence of a high dosage of chlorides in concrete doesn’t affect the compressive strength negatively^1,5,12,18^. However, there are some mixes showed the opposite (mixes no: 15–16-17–18) and this might be due to a slight error in curing or compaction in these mixes. For mixes containing MK, it was observed that there is no direct or clear relation between replacement level and compressive strength. This might be attributed to the non-uniformity in the properties of the clay used in MK formation, also the calcining temperature of the clay is so sensitive and has a high influence on the properties of the produced MK therefore, the MK used in this study may be not pozzolanic enough to increase the compressive strength or even to provide a clear relation between the replacement level and the compressive strength.

### Bond strength

**Figure 6:**
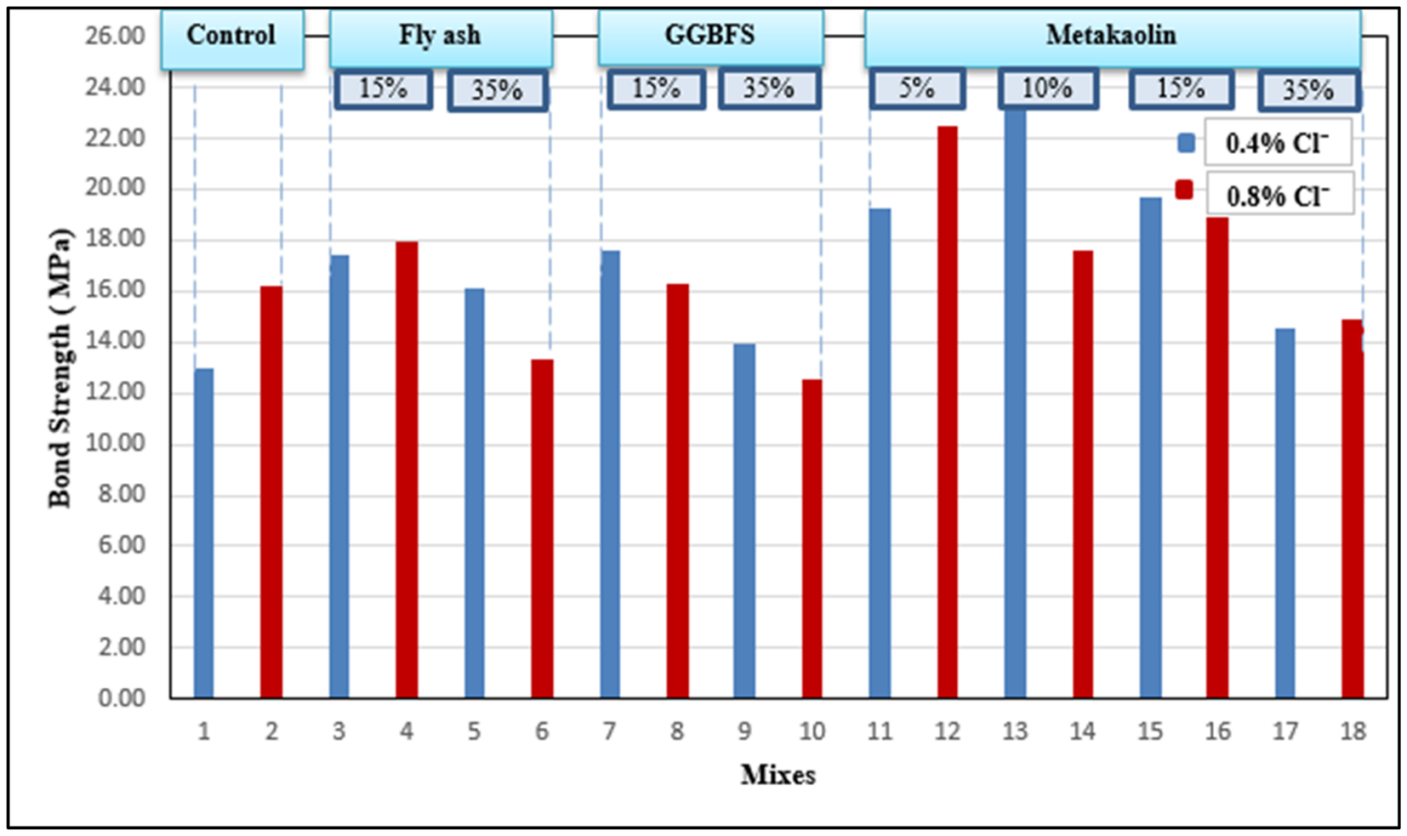
Bond strength of concrete mixes.

The pull-out test is implemented at age 28 days to investigate the bond strength of the hardened concrete mixes and the results can be shown in Fig. 6. The bond strength of mixes containing FA and GGBFS was found to be similar to control mixes containing OPC only while mixes containing MK had higher bond strengths (especially 5%MK and 10%MK). Although the variance among results is considered small, the results can be explained as follows: the bond strength mainly depends on cohesion, friction, and bearing. Adding pozzolanic materials contributed to the cohesion between paste and steel rebars which has a minimum effect on increasing the bond strength so, most of the mixes had bond strength similar to control mixes. Also, the increase in bond strength in some mixes containing MK can be referred to uncommon phenomenon as MK might work as a filler material like fine aggregate not a pozzolanic one which increases the friction and bearing between concrete and steel rebars leading to an increase in the bond strength.

**Figure 7:**
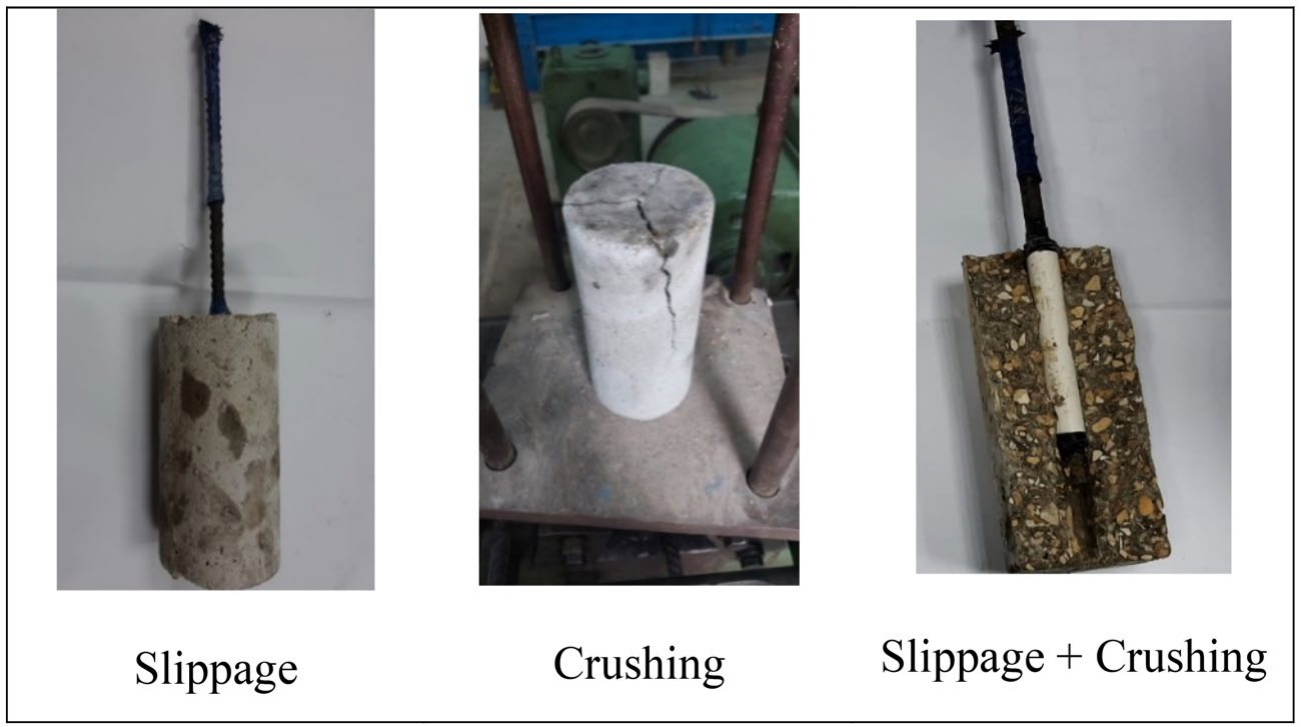
Failure modes of pull-out test.

The failure modes varied among mixes as some mixes failed by slippage of steel rebars, others failed by crushing of concrete, or slippage with crushing as shown in Fig. 7. For future research, it’s recommended to study the mechanical properties (including both compressive and bond strength) of such mixes in long-term time intervals to be able to assess the effect of the chloride binding process on these mechanical properties in later ages.

### Half-cell potential test

**Figure 8:**
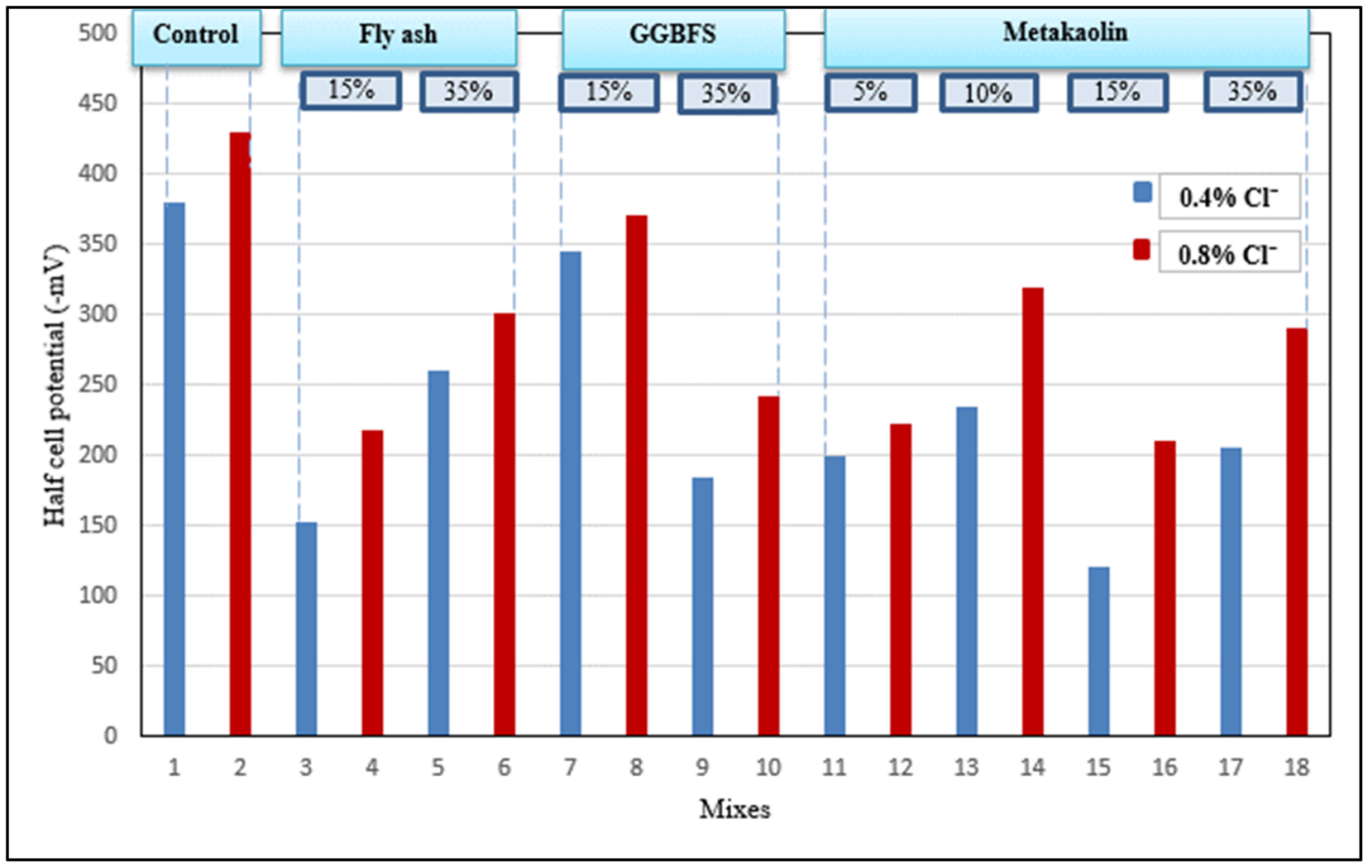
Half-cell potential test results.

This test was implemented after 28 days to determine the probability of reinforcement steel to corrode in each concrete mix^21,30^and test results are presented in Fig. 8.

As shown in Fig. 8, the corrosion potential of OPC mixes is the highest among all mixes which indicates that steel embedded in concrete mixed with OPC only is susceptible to corrosion much more than that embedded in concrete mixed with pozzolans as a partial replacement of OPC. Also, it’s clearly shown that mixes with 0.8% Cl^−^ have a higher corrosion potential than mixes with 0.4% Cl^−^ which sounds very logical. For mixes containing MK, the irregular changes in the corrosion potential can be attributed to the irregularity of the MK properties due to containing non-uniform clay or inadequate calcining temperature as explained previously in Sect. "Compressive strength".

Returning to the results of the chloride binding capacity test, it could be indicated that having high chloride binding capacity led to have low corrosion potential (35% GGBFS and 5%MK) which makes these mixes considered the best replacement levels regarding corrosion resistance. Also, this asserts that the chloride binding process has a vital role in reducing the probability of steel corrosion. Even mixes containing 0.8% Cl^−^ have potentials < − 350 mV which is considered the value above which (− 400, − 450,…) the steel becomes active and ready for corrosion.

### Accelerated corrosion test

**Figure 9:**
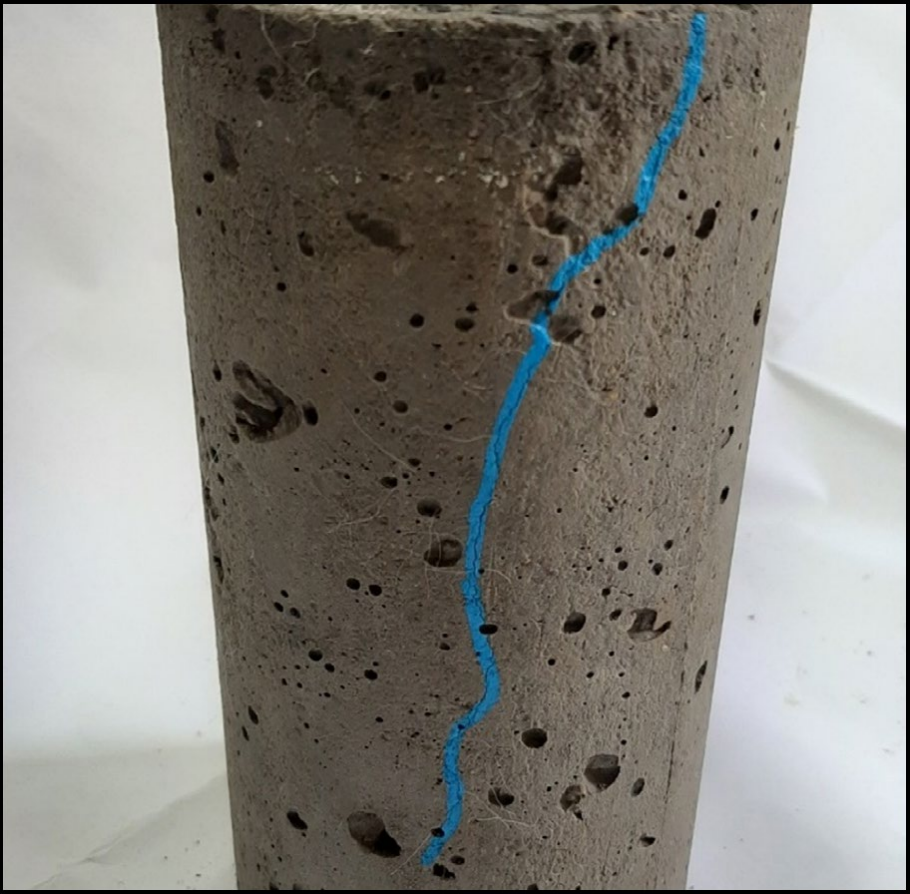
Concrete cracking due to steel corrosion.

The accelerated corrosion test was carried out to compare the corrosion behavior among the different concrete mixes^21,40^. All specimens were checked every day since the beginning of the test to observe and record the date of the cracking initiation which occurs because of the volumetric increase in the steel rebar which in turn causes internal compressive stresses on the concrete specimen consequently, it starts to crack ^30^ as shown in Fig. 9.

**Figure 10:**
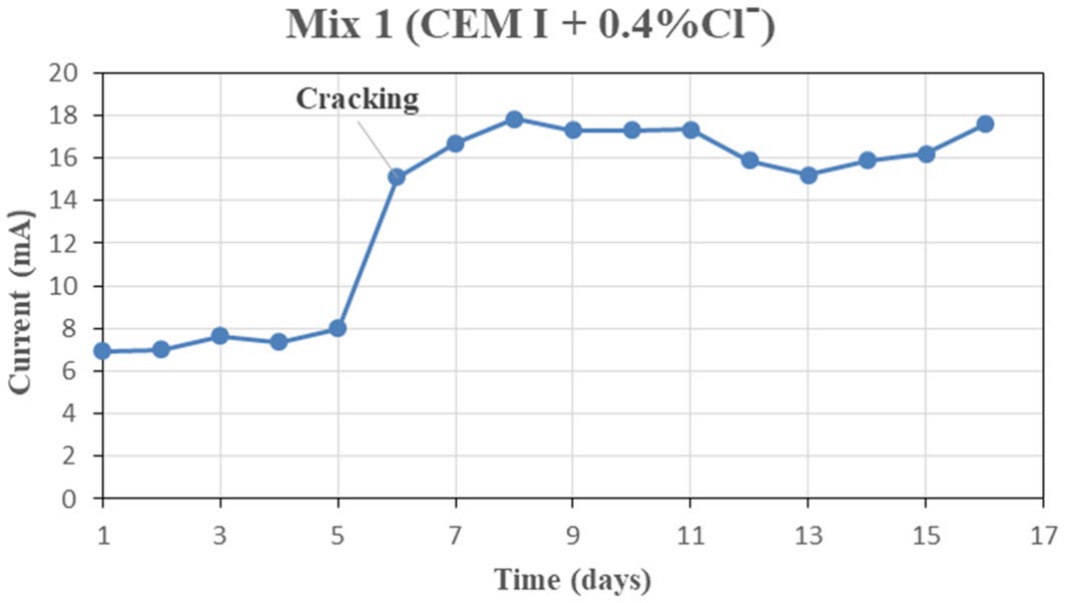
Current–time relation for mix 1.

For each concrete mix, the relation between electrical current in milliamperes and time in days was determined as shown in Fig. 10 as a typical one.

**Figure 11:**
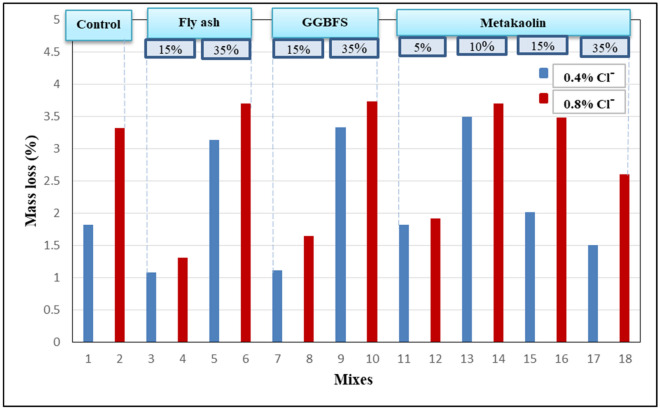
The mass loss of steel rebars after corrosion.

From the previous typical chart, the cracking time and the range of the electrical current for each concrete mix can be presented in Table 7. Also, the mass loss of the steel rebars due to corrosion was calculated from the weight of the steel rebars before and after corrosion. Results are presented in Fig. 11.

**Table 7:** Cracking time and range of electric current for each concrete mix.

Mix no		Cracking time (day)	Range of electric current (milli-ampere)
1	OPC-0.4%	6	7–18
2	OPC-0.8%	6	4–26
**3**	**15%FA-0.4%**	**47**	**2**–**10**
**4**	**15%FA-0.8%**	**42**	**3**–**10**
5	35%FA-0.4%	15	1–16
6	35%FA-0.8%	15	1–16
7	15%GGBFS-0.4%	4	10–22
8	15%GGBFS-0.8%	6	10–22
**9**	**35%GGBFS-0.4%**	**42**	**5**–**13**
**10**	**35%GGBFS-0.8%**	**29**	**1**–**16**
11	5%MK-0.4%	8	4–27
12	5%MK-0.8%	7	7–26
13	10%MK-0.4%	5	7–18
14	10%MK-0.8%	5	9–18
15	15%MK-0.4%	7	6–18
16	15%MK-0.8%	7	6–20
**17**	**35%MK-0.4%**	No cracking observed	**2**–**10.5**
**18**	**35%MK-0.8%**		**2**–**11**

Significant values are in [bold].

To analyze the previous results properly, it should be noted that the corrosion process in this test happens due to two main causes. The first one is the free chlorides existing in the concrete mix surrounding steel rebar, so, to hinder these free chloride ions from reaching the surface of steel rebar, the cementitious material used in the concrete must have a high chloride binding capacity. The other cause of corrosion is the external solution of sodium chloride and to prevent the transportation of such external chlorides to steel rebar through concrete, the concrete must be of high quality having a dense microstructure and this is reachable by adding mineral admixtures that have high pozzolanic reactivity ^30^.

So, mixes having high chloride binding capacity with high pozzolanic reactivity are considered ideal to resist corrosion in this test. Mixes 9 and 10 (35%GGBFS) set an example for such mixes ^30^. For mixes having low chloride binding (more free chlorides) like control mixes, and 15%GGBFS we can see that they have low resistance against corrosion as they cracked at an early age relatively. Also, mixes 11–16 (5%MK–15%MK) cracked early as they have low pozzolanic reactivity so, they were easily penetrated by external chlorides while mixes 17 and 18 (35%MK) had better corrosion resistance as the MK precipitated in the pores and contributed to filling the voids and densifying the microstructure of the concrete significantly which in turn led to hindering the external chlorides from transporting through concrete therefore, the high corrosion resistance of theses mixes doesn’t contradict with the low chloride binding capacity of them.

For mass loss results, it must be noted that making an adequate comparison between results requires that the testing time must be fixed for all mixes which had not happened, so the results are not expressive as they were intended to be.

### Rapid chloride penetration test

**Figure 12:**
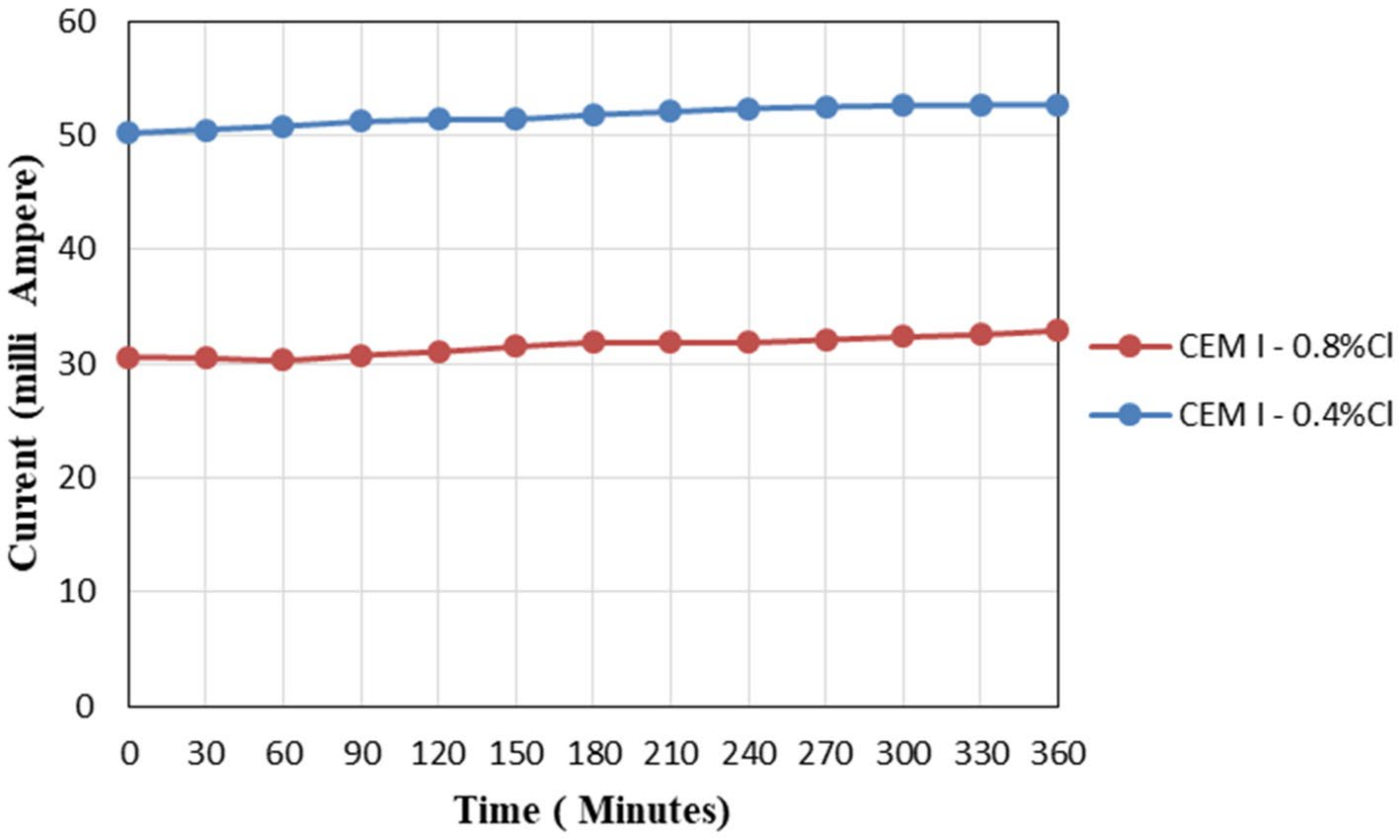
RCPT curves for control mixes.

RCPT was conducted for each concrete mix to determine its electrical conductivity and, therefore, its permeability. The relation between current in milliamperes and time in minutes can be presented in Fig. 12 (typical figure) and the test results can be summarized in Table 8.

**Table 8:** RCPT results.

Mix no		Charge passed (Coulombs)	Chloride ion penetrability
1	OPC-0.4%	1117	Low
2	OPC-0.8%	681	Very low
3	15%FA-0.4%	1412	Low
4	15%FA-0.8%	1316	Low
5	35%FA-0.4%	793	Very low
6	35%FA-0.8%	636	Very low
7	15%GGBFS-0.4%	1186	Low
8	15%GGBFS-0.8%	586	Very low
9	35%GGBFS-0.4%	1528	Low
10	35%GGBFS-0.8%	1320	Low
11	5%MK-0.4%	629	Low
12	5%MK-0.8%	628	Very low
13	10%MK-0.4%	1778	Low
14	10%MK-0.8%	1628	Low
15	15%MK-0.4%	1126	Low
16	15%MK-0.8%	869	Very low
17	35%MK-0.4%	311	Very low
18	35%MK-0.8%	359	Very low

As indicated in the results, all concrete mixes showed an acceptable performance according to chloride ion permeability which indicates that reinforced concrete mixed with SCMs has a high resistance against corrosion caused by transporting external chlorides through concrete reaching the steel surface. Also, it is confirmed by many researchers that the process of chloride binding leads to enhancing the concrete resistance against chloride diffusion from the outer environment^2,4,10,13,15,32^.

### Sorptivity test

**Figure 13:**
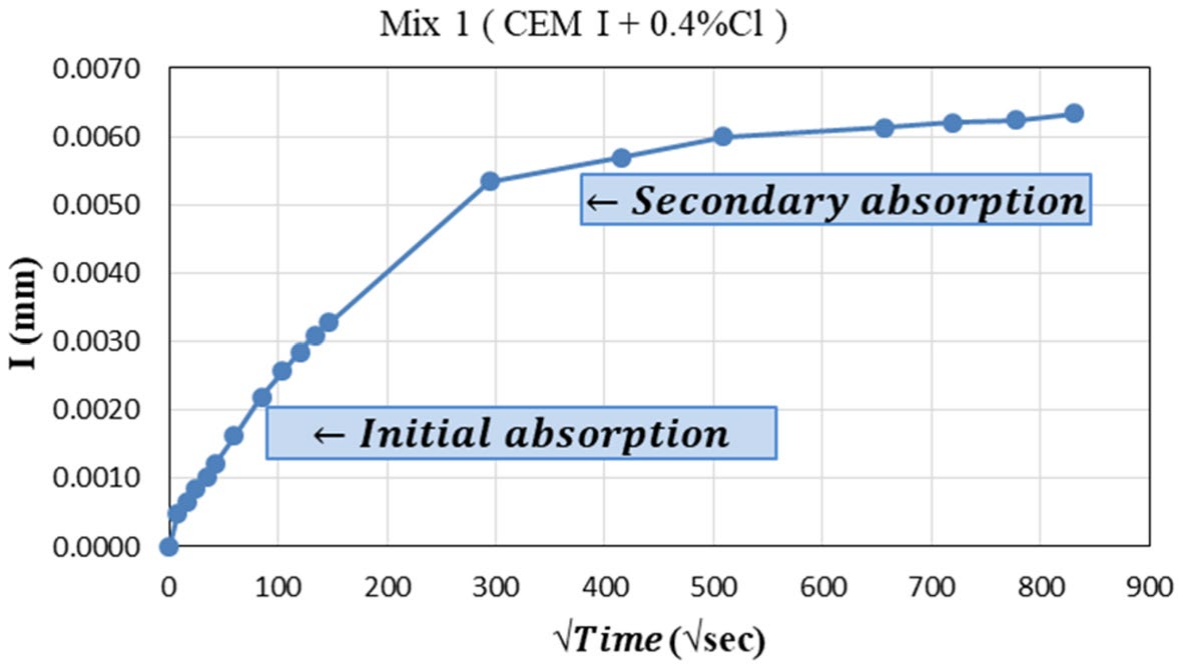
Current–time relation for mix 1.

This test was implemented to provide a good indicator of concrete durability. Figure 13 shows the relation between absorption and time for mix 1 which has the same trend as all the other mixes. The rate of initial and second absorption is calculated as the slope of the first and second lines in the curve respectively. The test results are summarized in Table 9.

**Table 9:** Sorptivity test results.

Mix no		Rate of initial absorption (mm/sec0.5)	Rate of secondary absorption (mm/sec0.5)	Max. value of absorption *I* (mm)
1	OPC-0.4%	2.26 × 10^−5^	1.06 × 10^−6^	0.0063
2	OPC-0.8%	0.88 × 10^−5^	1.72 × 10^−6^	0.0026
3	15%FA-0.4%	1.05 × 10^−5^	1.72 × 10^−6^	0.0048
4	15%FA-0.8%	2.03 × 10^−5^	1.86 × 10^−6^	0.0083
5	35%FA-0.4%	2.51 × 10^−5^	1.55 × 10^−6^	0.0082
6	35%FA-0.8%	2.37 × 10^−5^	1.72 × 10^−6^	0.0084
7	15%GGBFS-0.4%	2.59 × 10^−5^	1.15 × 10^−6^	0.0076
8	15%GGBFS-0.8%	2.16 × 10^−5^	2.30 × 10^−6^	0.0078
9	35%GGBFS-0.4%	1.80 × 10^−5^	1.72 × 10^−6^	0.0086
10	35%GGBFS-0.8%	3.31 × 10^−5^	5.75 × 10^−7^	0.0098
11	5%MK-0.4%	1.94 × 10^−5^	2.30 × 10^−6^	0.0061
12	5%MK-0.8%	1.94 × 10^−5^	2.30 × 10^−6^	0.0076
13	10%MK-0.4%	1.73 × 10^−5^	6.21 × 10^−7^	0.0053
14	10%MK-0.8%	1.42 × 10^−5^	1.55 × 10^−6^	0.0084
15	15%MK-0.4%	1.29 × 10^−5^	1.80 × 10^−6^	0.0043
16	15%MK-0.8%	1.80 × 10^−5^	9.32 × 10^−7^	0.0053
17	35%MK-0.4%	1.65 × 10^−5^	1.24 × 10^−6^	0.004
18	35%MK-0.8%	2.73 × 10^−5^	1.24 × 10^−6^	0.0059

### pH test

**Figure 14:**
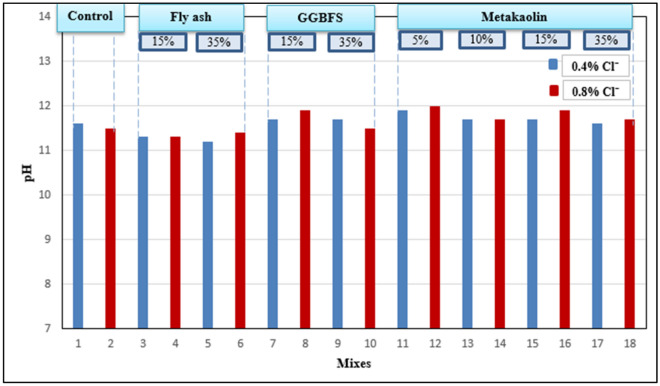
pH values of concrete mixes.

The pH value of each concrete mix was measured and the results are presented in Fig. 14. As shown in the results, the pH values of all mixes are somehow similar and they ranged from 11.2 to 12. Results also show that adding pozzolans with a high dosage of salts into concrete mixes doesn’t affect the pH value badly and the pH values in all mixes are above the limit behind which the corrosion may happen^9^.

### XRD

**Figure 15:**
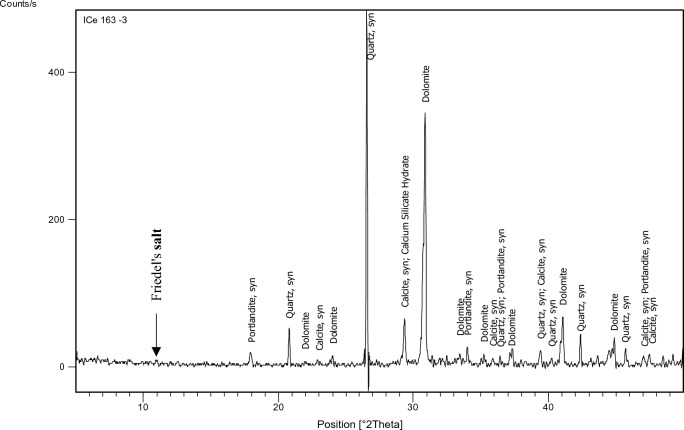
XRD analysis for mix 1 (OPC).

**Figure 16:**
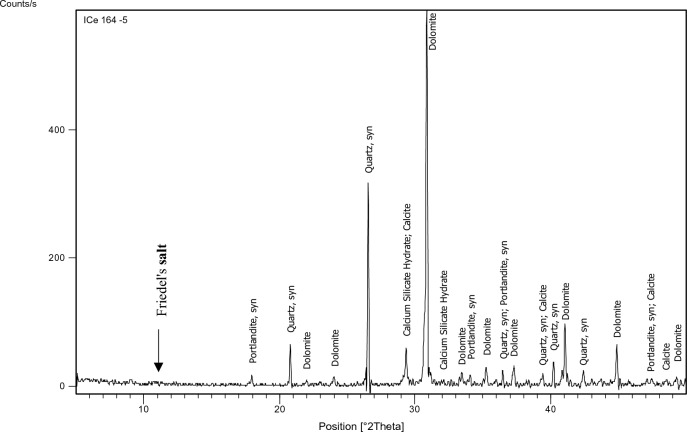
XRD analysis for mix 3 (15% FA).

**Figure 17:**
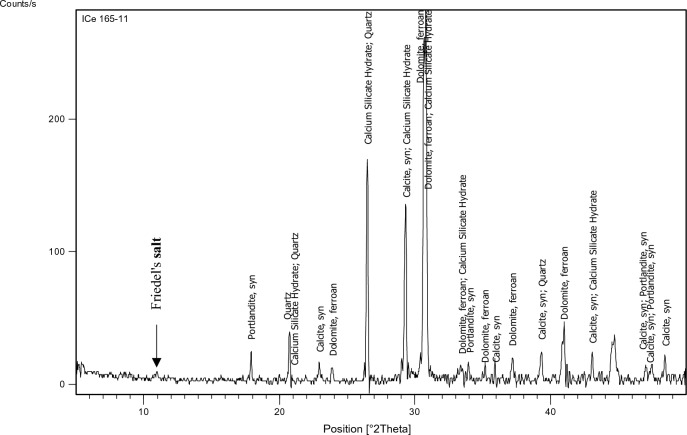
XRD analysis for mix 7 (15% GGBFS).

**Figure 18:**
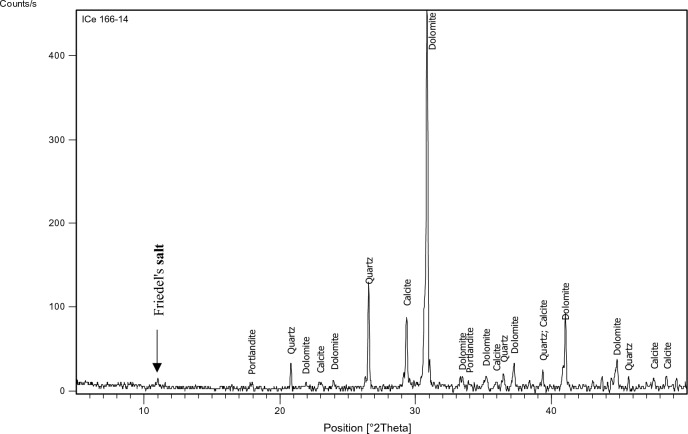
XRD analysis for mix 9 (35% GGBFS).

**Figure 19:**
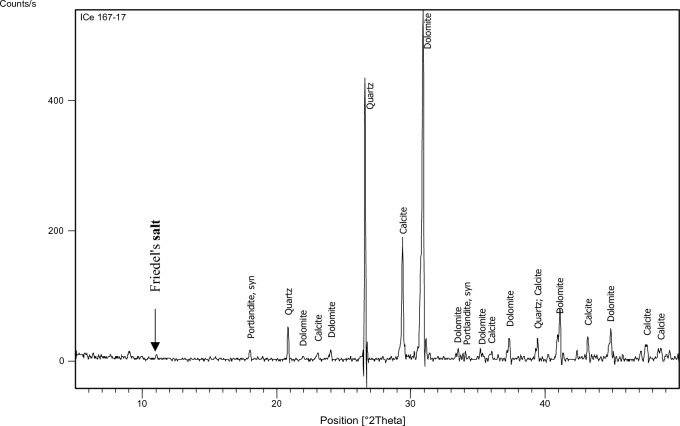
XRD analysis for mix 11 (5% MK).

**Figure 20:**
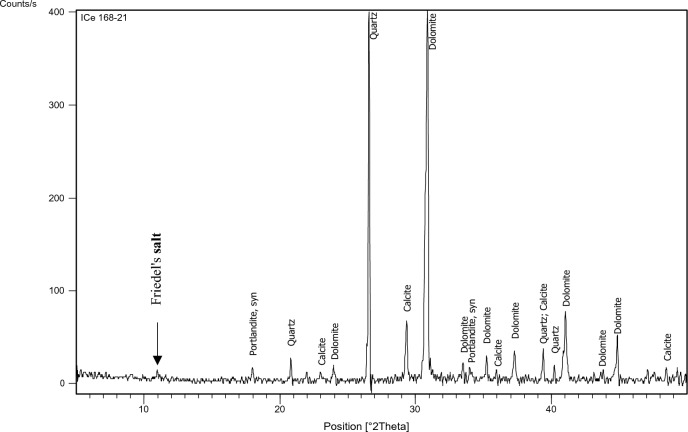
XRD analysis for mix 15 (15% MK).

Figures 15, 16, 17, 18, 19 and 20 present the XRD results for different mixes that contain 0.4%Cl^−^.

The XRD analysis shows that the quantity of Friedel’s salt in mix 9 (35%GGBFS-0.4%Cl) is the highest among the other mixes which is compatible with the results of the chloride binding capacity. While its amount in OPC can be considered the least.

## Conclusions

From the results of the experimental program carried out in this research work, the following points can be concluded:

- The chloride binding capacity of SCMS (FA, GGBFS, and MK) is better than OPC which has a significant effect on lowering the risk of corrosion of the reinforcement steel embedded in the concrete.
- In this study, the best replacement levels of OPC are 35%GGBFS and 5%MK as they show the highest chloride binding capacity and lowest corrosion potential with accepted mechanical and durability properties.
- For concrete mixed with 35%GGBFS or 5%MK as a partial replacement of OPC, the limit of chloride content may be increased to 0.5% from the binder weight instead of 0.4% without causing an increase in the probability of steel corrosion.
- In Chemical chloride binding, the binders with low content of CaO have a lower binding capacity as calcium oxide is also needed to form Friedel’s salt like aluminum oxide.
- All concrete mixes including SCMs as a partial replacement of OPC have a lower corrosion probability than mixes containing OPC only.
- Utilizing a chloride content of a percentage of 0.8% from the binder weight in concrete mixes doesn’t have an obvious negative effect on both mechanical and durability properties.
- The concrete mix should be designed based on its ambient environment. For reinforced concrete intended to be poured with ingredients containing chlorides, the main concern in choosing the type and the percentage of the binder is to have a high binding capacity. But for reinforced concrete intended to be located adjacent to external sources of chlorides, the main concern is to choose a binder that has a high pozzolanic reactivity to densify and enhance the microstructure of the concrete preventing external chlorides from penetrating concrete and reaching the steel surface.

## Recommendations for further work

- New allowable limits of chloride content in reinforced concrete should be set for reinforced concrete containing binders other than OPC because every binder type or percentage has a different binding capacity.
- Further research is needed to study the chloride binding capacity of geopolymer concrete especially that based on GGBFS as the increase in the replacement level in this work has led to an increase in the binding capacity.
- Further research is needed to investigate the chloride binding process in the long term and to clarify the relation between concrete age and both physical and chemical binding.
- Also, further studies are needed to show the relation between the external chlorides that may penetrate the concrete and the binding process happening inside.

## Data Availability

Data will be made available on request. To request any data please contact heba.abdelfattah.94@eng.asu.edu.eg or m.kohail@eng.asu.edu.eg.

## Abbreviations

OPC: Ordinary Portland Cement
SCMs: Supplementary cementitious materials
GGBFS: Ground granulated blast furnace slag
FA: Fly ash
MK: Metakaolin
Cl^−^: Chloride ions
OH^−^: Hydroxide ions
C_3_A: Tri calcium aluminate
CBC: Chloride binding capacity
XRD: X-ray diffraction
RCPT: Rapid chloride penetration test
DC: Direct current
